# Differential Alteration in Expression of Striatal GABA_A_R Subunits in Mouse Models of Huntington’s Disease

**DOI:** 10.3389/fnmol.2017.00198

**Published:** 2017-06-20

**Authors:** Zhuowei Du, Margot Tertrais, Gilles Courtand, Thierry Leste-Lasserre, Laura Cardoit, Frédérique Masmejean, Christophe Halgand, Yoon H. Cho, Maurice Garret

**Affiliations:** ^1^Institut de Neurosciences Cognitives et Intégratives d’Aquitaine, UMR 5287, University of BordeauxBordeaux, France; ^2^Centre National de la Recherche Scientifique, Institut de Neurosciences Cognitives et Intégratives d’Aquitaine, UMR 5287Bordeaux, France; ^3^Institut National de la Santé et de la Recherche Médicale, Neurocentre Magendie, U862, Physiopathologie de la Plasticité NeuronaleBordeaux, France; ^4^Neurocentre Magendie, Physiopathologie de la Plasticité Neuronale, U862, University of BordeauxBordeaux, France

**Keywords:** Huntington’s disease, R6/1 mouse model, HdhQ111, striatum, GABA_A_ receptor, synapse, parvalbumin interneuron, cholinergic interneuron

## Abstract

Huntington’s disease (HD) is a neurodegenerative disorder characterized by progressive motor symptoms that are preceded by cognitive deficits and is considered as a disorder that primarily affects forebrain striatal neurons. To gain a better understanding of the molecular and cellular mechanisms associated with disease progression, we analyzed the expression of proteins involved in GABAergic neurotransmission in the striatum of the R6/1 transgenic mouse model. Western blot, quantitative PCR and immunohistochemical analyses were conducted on male R6/1 mice and age-matched wild type littermates. Analyses were performed on 2 and 6 month-old animals, respectively, before and after the onset of motor symptoms. Expression of GAD 67, GAD 65, NL2, or gephyrin proteins, involved in GABA synthesis or synapse formation did not display major changes. In contrast, expression of α1, α3 and α5 GABA_A_R subunits was increased while the expression of δ was decreased, suggesting a change in tonic- and phasic inhibitory transmission. Western blot analysis of the striatum from 8 month-old Hdh Q111, a knock-in mouse model of HD with mild deficits, confirmed the α1 subunit increased expression. From immunohistochemical analyses, we also found that α1 subunit expression is increased in medium-sized spiny projection neurons (MSN) and decreased in parvalbumin (PV)-expressing interneurons at 2 and 6 months in R6/1 mice. Moreover, α2 subunit labeling on the PV and MSN cell membranes was increased at 2 months and decreased at 6 months. Alteration of gene expression in the striatum and modification of GABA_A_ receptor subtypes in both interneurons and projection neurons suggested that HD mutation has a profound effect on synaptic plasticity at an early stage, before the onset of motor symptoms. These results also indicate that cognitive and other behavioral deficits may be associated with changes in GABAergic neurotransmission that consequently could be a relevant target for early therapeutic treatment.

## Introduction

Huntington’s disease (HD) is a neurodegenerative disease characterized by a progressive decline of motor capabilities and cognitive functions. Animal models and a few post-mortem studies have shown only limited signs of neuron loss in the brain, despite overt clinical symptoms of the disease, suggesting that neuronal and synaptic dysfunction, rather than cell death, may underlie early behavioral manifestations of the disease (Mizuno et al., 2000; Caramins et al., 2003; Raymond et al., 2011; Morton, 2013; Du et al., 2016).

Huntington’s disease is caused by a dominant mutation of the gene that encodes the huntingtin protein. The expression of the huntingtin protein is ubiquitous, and the mutation affects virtually all brain structures. However, alterations are most obvious in the striatum (Francelle et al., 2014). This structure is the main input nucleus of the basal ganglia and is involved in cognitive and motivational functions in addition to motor control (Tepper et al., 2007). The predominant local interneurons and projection neurons (medium spiny neurons, MSNs) in the striatum are GABAergic. It is well established that the GABAergic control of MSNs originates from parvalbumin-, calretinin-, somatostatin, or nNOS-expressing interneurons (INs) as well as collaterals from MSNs (Gittis and Kreitzer, 2012). In addition to these GABAergic neurons, the striatum also contains cholinergic INs that regulate local inhibitory circuits (English et al., 2012). In addition to this established circuitry, very recent findings suggest a more complex GABAergic control of MSNs in the striatum through GABA release from midbrain dopaminergic neurons or GABAergic external globus pallidus (GPe) neurons (Mallet et al., 2012; Tritsch et al., 2012; Glajch et al., 2016). Several studies have described a functional alteration of striatal GABA activity in HD (Cummings et al., 2010; Cepeda et al., 2013; Wojtowicz et al., 2013). However, an alteration in striatal GABAergic signaling in HD at the molecular level is essentially unknown.

Diversity in GABAergic signaling is due to several pre- and post-synaptic factors that are the target of many drugs that are currently in wide clinical use (Mohler, 2011). It is well documented that an alteration in any aspect of this system is linked to several neurological and neurodevelopmental disorders (Braudeau et al., 2011; Luscher et al., 2011; Brickley and Mody, 2012; Rudolph and Mohler, 2013; Braat and Kooy, 2015). Post-synaptic GABA_A_Rs are of particular interest because a modification in their subunit expression can dramatically alter GABAergic signaling. GABA_A_Rs are oligomeric proteins composed of α-, β-, and γ- (or δ-) subunits (Olsen and Sieghart, 2009). Strong evidence supports a model in which subunit composition confers a distinctive cellular distribution, functional properties, and the specific effect of allosteric modulators like benzodiazepines or neurosteroids (Olsen and Sieghart, 2009; Luscher et al., 2011). In brain regions, including the striatum, synaptic neurotransmission mediating phasic inhibition is linked to GABA_A_Rs composed of α1, α2, or α3 in combination with β and γ2 subunits. The substitution of an α5 for an α1-3 or a δ for a γ2 subunit has been shown to form extrasynaptic receptors mediating tonic inhibition (Luo et al., 2013; Ferando and Mody, 2014). Indeed, it is now established that receptor subtypes are associated with significant physiological outcomes and specific cognitive functions (Rudolph et al., 1999; Rudolph and Mohler, 2013).

Huntington’s disease progression and symptoms are recapitulated in transgenic mouse models (Pouladi et al., 2013) such as the R6/1 mouse line that is widely used because of adult onset, slow disease progression and cognitive deficits beginning at 3 months of age followed by motor impairment (Milnerwood et al., 2006; Pang et al., 2009; Crook and Housman, 2011; Lebreton et al., 2015). Moreover, various genome-wide gene expression analyses in the striatum of human and mouse models, including R6/1, have shown that a number of differentially expressed genes are involved in neurotransmission (Kuhn et al., 2007; Seredenina and Luthi-Carter, 2012; Achour et al., 2015; Langfelder et al., 2016). To assess the extent of GABAergic system alterations in HD, we analyzed the expression of genes involved in striatal GABAergic neurotransmission. Specifically, we used the R6/1 mouse model at 2 months of age when animals are defined as motor pre-symptomatic and at 6 months when they are fully symptomatic.

## Materials and Methods

Male R6/1 transgenic mice and age-matched male wild type littermates, obtained by crossbreeding male R6/1 (C57/BL6 background) and female C57/BL6 animals (Pietropaolo et al., 2014), were used in our experiments. This R6/1 line expresses exon 1 of the human huntingtin gene with an expanded 116–126 CAG trinucleotide repeats. Male *HdhQ111* knock-in mice in a CD1 background, with targeted insertion of a chimeric human–mouse exon 1 with 109 CAG repeats and the corresponding *WT* littermates, were also used (Wheeler et al., 2002). All animals were genotyped by PCR with DNA extracted from tail specimens. The animals were housed with 12–12 h light-dark cycle (light on at 8 a.m.) with unlimited food/water access. Animal maintenance and experiments were approved by the Institutional Animal Care and Use Committee (# A50120127) and were carried out in accordance with the European Communities Council Directive 2010/63/EU for animal experiments.

### Western Blot

Three hundred and fifty micrometer acute slices from 3 to 4 animals of the same age and genotype were prepared to dissect out and pool the striatum under a Nikon SMZ800 bright field microscope with a SCHOTT KL1500 LCD illuminator (Du et al., 2016). RIPA buffer (Sigma-Aldrich, R0278) with a protease inhibitor cocktail (Roche Diagnostics, 11836153001) was used for protein extraction at a ratio of 20 μL per mg of tissue. The brain tissues were disrupted and homogenized by a sonicator (Ultrasons, Annemasse, France) on ice. The homogenates were centrifuged (1–15k, Sigma) at 13000 rpm for 30 min at 4°C. The supernatants were collected as total cell lysates. Protein concentration was measured with a RC DC^TM^ protein assay kit (BIO-RAD) according to manufacturer instructions. Samples were divided into 15 μg protein aliquots and kept frozen until used. Samples were supplemented with loading buffer, proteins were separated on SDS-PAGE and revealed by Western blotting using specific antibodies (**Table 1**), and analyzed as previously described (Chaumont et al., 2013). Briefly, protein bands were detected using an ECL chemiluminescence detection system (Lumiglo/Eurobio) with autoradiography film (Amersham Hyperfilm^TM^ ECL). Bands were quantified using ImageJ software, and the results obtained for each sample were normalized to the amount of GAPDH measured. The mean value in the WT littermate group, which was processed in parallel, was taken as 100%. Note that, as specified in the corresponding figure legends, each data set is representative of four independent western blot analyses. Moreover, individual analyses were performed with pooled brain structures from at least three mice of the same age and genotype. Therefore, we believe that our data represent the average protein expression of the different groups of mice and avoids individual variability.

**Table 1:** List of antibodies used for Western blot (WB) and immunohistochemistry (IHC).

Target	Distributor	Catalog #	Dilution	Application	Reference, Specificity
GABA_A_R α1	Alomone NeuroMab	AGA-001N95/35	1:30001:2500	IHC & WB (or IHC/WB)	(Chaumont et al., 2013)
GABA_A_R α5	Synaptic Systems	224 503	1:2000	WB	(Brady and Jacob, 2015)
GABA_A_R β2	AbCys	VPA 5561	1:1000	WB	(Du et al., 2016)
GABA_A_R β3	NeuroMab	N87/25	1:1000	WB	(Gurba et al., 2012)
GABA_A_R γ2	Alomone	AGA-005	1:300	WB	(Vlachos et al., 2013)
GABAAR δ	Millipore	AB9752	1:1000	WB	(Sarkar et al., 2011)
Vgat	Synaptic Systems	131011	1:1500	WB	(Fidzinski et al., 2015)
NeuN	Chemicon	MAB377	1:500	IHC	(Kang et al., 2012)
DARPP-32	BD biosciences	611520	1:1000	IHC	(Pietropaolo et al., 2014)
ChAT	Millipore	AB144P	1:400	IHC	(Deshpande et al., 2013)
Parvalbumin	Synaptic Systems	195004	1:1000	IHC	(Reichel et al., 2014)
Gephyrin	Synaptic Systems	147003	1:5000	WB	(Winkelmann et al., 2015)
GAD6	Chemicon	AB1511	1:4000	WB	(Yeo et al., 2010)
GAD67	Synaptic Systems	198011	1:2500	WB	(Mikhaylova et al., 2014)
GAD65	Synaptic Systems	198103	1:4000	WB	(Saliba et al., 2009)
GAPDH	Abcam	6C5	1:100000	WB	(Chaumont et al., 2013)
VMaT2	Synaptic Systems	131011	1:1000	WB	(Brunk et al., 2006)
Calretinin	P. Ciofi lab	rabbit	1:2000	IHC	(Ciofi et al., 2006)
Somatostatin	P. Ciofi lab	rabbit	1:1500	IHC	(Ciofi et al., 2006)
Neuroligin2	F. Varoquaux lab	rabbit	1:2000	WB	(Poulopoulos et al., 2009)

### Quantitative Real-Time PCR (q-PCR)

Tissue samples were homogenized in Tri-reagent (Euromedex, France) and RNA was isolated using a standard chloroform/ isopropanol protocol (Chomczynski and Sacchi, 1987). RNA was processed and analyzed following an adaptation of published methods (Bustin et al., 2009). cDNA was synthesized from 2 μg of total RNA using RevertAid Premium Reverse Transcriptase (Fermentas) and primed with oligo-dT primers (Fermentas) and random primers (Fermentas). q**-**PCR was performed using a LightCycler^®^ 480 Real-Time PCR System (Roche, Meylan, France). q**-**PCR reactions were made in duplicate for each sample, using transcript-specific primers, cDNA (4 ng) and LightCycler 480 SYBR Green I Master (Roche) at a final volume of 10 μl. The PCR data were exported and analyzed using a software tool (Gene Expression Analysis Software Environment) developed at the NeuroCentre Magendie. For determination of the reference gene, the Genorm method was used (Bustin et al., 2009). We analyzed multiple reference genes for normalization of the q-PCR data: glyceraldehyde-3-phosphate dehydrogenase (Gapdh), actin-beta (Actb), a hydroxymethylbilane synthase (Hbms) ribosomal protein L13a (Rpl13a) succinate dehydrogenase complex, subunit A (Sdha), ubiquitin C (Ubc), tyrosine 3-monooxygenase/tryptophan 5-monooxygenase activation protein, zeta (Ywhaz) eukaryotic translation elongation factor 1 alpha 1 (Eef1a1), peptidylprolyl isomerase A (Ppia), tubulin, alpha 4a (Tuba4a), non-POU domain containing, octamer-binding (Nono) glucuronidase, beta (Gusb). Relative expression analysis was corrected for PCR efficiency and normalized against Gapdh and Ppia genes that were the most stably expressed. The relative level of expression was calculated using the comparative (2^-ΔΔCT^) method (Livak and Schmittgen, 2001). Primer sequences are reported in **Table 2**.

**Table 2:** Mouse q-PCR primer sequences.

Gene	GenBank ID	Forward Sequence (5′–3′)	Reverse Sequence (5′–3′)
Gapdh	NM_008084	TCAAGAAGGTGGTGAAGCAG	TGGGAGTTGCTGTTGAAGTC
Ppia	NM_008907	CAAATGCTGGACCAAACACAA	GCCATCCAGCCATTCAGTCT
Gabra1	NM_010250	AGAAGTCTGTGGCCCACAACA	CAGCAGAGTGCCATCCTCTGT
Gabrd	NM_008072	AGGTGGTTGCCACAAACTCCT	AAGCCGAGCCTCCTCTCTGT
Gad67	NM_008077	GACCAATCTCTGTGACTCGCTTAG	CTGGTCAGTGTATCGGAGGTCTT
Gabrb1	NM_008069	CCTCGCAGCTCAAAGTGAAGA	GAACATTCGGGACCACTTGTCT
Gabrb2	NM_008070	CCCACCTCCGGGAAACTC	GAAGACAAAGCACCCCATTAGG
Gabrb3	NM_008071	AAAGGATCGAGCTCCCACAGT	TGTGGCGAAGACAACATTCC
Gabrg1	NM_010252	GTTGCCAATGCTACATCTGTGAG	AGCCATCTTGCCAGACCTACA
Gabrg2	NM_177408	TCCαAGGCTGATGCTCACT	ACTCGACCATCATTCCAAATTCTC
Gabra2	NM_008066	CACAATGGAGAGCAGCTTACCA	GCTCAGGATGGGTACAGAAGTGT
Gabra3	NM_008067	CAAAGGTαGCCACAAATAGCAC	CCTTAGGAGCCTTGCTCAGTGA
Gabra4	NM_010251	TATCAAAGCCTCCCCCAGAAGT	CTGAAGGGATGTTTCTGTGTGTTT
Gabra5	NM_176942	TTTTAGGGAACCCCTGTGATGA	TTAACAGCGTGTACCCCAGGA
VGAT	NM_009508	GCTTGGAAACTTGACCTTGAGG	ACGCTGTAGATTCCAAGCACTG
GAD65	NM_008078	CATGGTCATCTCAAACCCTGC	CGAGGCGTTCGATTTCTTCA
Geph	NM_172952	GACAGAGCAGTACGTGGAACTTCA	GTCACCATCATAGCCGTCCAA
Nlgn2	NM_198862	TCCCCCATGGAAATGTAGTTCT	CATGGCTGTTCCAAGAGTTGC

### Immunohistochemistry

Animals were anesthetized with ketamine/xylazine and perfused transcardially with ice-cold modified artificial cerebrospinal fluid (ACSF), equilibrated with 95% O_2_–5% CO_2_, and containing (in mM): 230 sucrose, 26 NaHCO_3_, 2.5 KCl, 1.25 NaH_2_PO_4_, 0.5 CaCl_2_, 10 MgSO_4_, and 10 glucose. After removal, brains were submerged in ice-cold modified ACSF and sectioned in 350 μm thick slices with a vibratome (VT1200S; Leica Microsystems, Germany) in the parasagittal plane. Slices were fixed in 4% paraformaldehyde for 30 min and processed into 16 μm-thick sections collected on gelatin-coated slides as previously described (Moragues et al., 2002; Schneider Gasser et al., 2006). Slices from the same hemisphere were cut serially in 16 μm thick sections (60–80 sections for one hemisphere) and placed on gelatin coated slides, one section per slide. All sections were stored at -80°C until used. Labeling were carried out on brains obtained from 3 mice for each age and each genotype.

After 1 h incubation in blocking solution containing phosphate-buffered saline (PBS), pH 7.4, 4% donkey serum and 0.3% Triton X-100 (Sigma), slides were incubated overnight at room temperature in primary antibodies (**Table 1**) diluted with the blocking solution. They were then rinsed in PBS and incubated for 1 h in blocking solution containing a cocktail of secondary antibodies conjugated to fluorescent probes (Alexa-488, -568, or -647-conjugated donkey anti-mouse, -rabbit, -guinea pig, or goat antibody; Jackson Immunoresearch). Sections were washed and coverslipped. For quantification of α1 subunit in PV cells and the neuropil, PV was labeled with visible-wavelength emission (Alexa Fluor 488) and α1 with far-red-emitting dye invisible to the naked eye (Alexa Fluor 647). For each animal, 8 striatal serial sections (one of every 10 sections) were taken for labeling and analysis. Observation and acquisition were performed with a Leica DM6000B microscope (Leica Microsystems, Mannheim, Germany) equipped with a Qimaging RETIGA EXI camera. PV cells were identified by eye and imaged. PV labeling was used to draw the outline of the cell and define a region of interest (width 2 μm) including the cell periphery and surrounding external neuropil (**Figure 6E**). Mean fluorescence intensity of α1 labeling was then automatically measured using a custom-made macro in ImageJ software. The background was centered on the cell containing the nucleus, where labeling of GABA_A_R subunits is assumed to be negative. Quantification was performed on 129 to 143 neurons per group from 3 independent experiments on brains obtained from three mice for each age and each genotype.

Double-labeling of α2- and a3-subunits on MSNs, PV-, nNOS-, and ChAT-positive interneurons, were performed as above and imaged with a BX51 Olympus Fluoview 500 confocal microscope using an oil-immersion 60 × objective and 1.4 numerical aperture. DARPP32, PV, nNOS or ChAT labeling was used to identify each neuronal cell type and to draw a 0.2 μm wide band that outlined identified cells and define regions of interest. Quantification of the number of receptor clusters on the cell periphery were performed using a custom-made macro in ImageJ software. During the analysis, an image in a region of interest was subjected to moment preserving thresholding, and clusters above 0.064 μm^2^ were counted. Counts were carried out on brains obtained from three mice for each age and each genotype.

### Statistical Analyses

For Western blot, q-PCR, acetylcholinesterase expression and striatum volume analyses, unpaired two-tailed Student’s *t*-test was applied to compare differences between two groups. Data are expressed as mean ± standard error of the mean (SEM) and were analyzed statistically with Graph-Pad Prism (GraphPad Software Inc.). “n” indicates the number of independent biological replicates used in each group. In image analyses, test of normality (Shapiro test) and analysis of equal variances (Bartlett-Levene test) were performed. When the two groups expressed a normal distribution (Shapiro test) and had the same variance (Bartlett-Levene test), a two-tailed *t*-test was applied. In other cases, a Mann–Whitney *U* test was used. The significance level of the tests (*p*-value) was 5%. ^∗^*p* < 0.05, ^∗∗^*p* < 0.01, ^∗∗∗^*p* < 0.001.

## Results

### The Expression of Proteins Involved in GABAergic Neurotransmission Is Altered in R6/1 Mice

**FIGURE 1:**
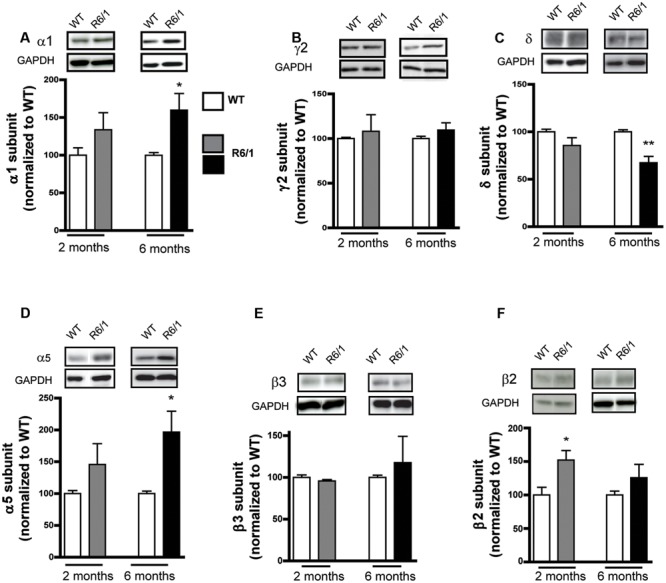
Western blot analysis of the striatum from 2 and 6 month-old mice. Membrane preparations extracted from the striatum were subjected to SDS-polyacrylamide gel electrophoresis and immunoblotted. Western blots were visualized with GABA_A_R subunit antibodies. **(A)** Representative Western blots (each out of four) showing α1 subunit immunoreactivity (top panels). The blots were also probed for GAPDH as a loading control. The bar graphs (lower panel) depict the means of four independent experiments showing α1 subunit expression levels after normalization to the corresponding expression of GAPDH. Each mean ratio for R6/1 mice (gray bar, 2 months; black bar, 6 months) is displayed as a percentage of the corresponding wild type value (WT, unfilled bars) set at 100%. **(B)** Expression of the γ2 subunit in the striatum at 2 months (*n* = 4) and at 6 months in R6/1 mice (*n* = 4). **(C)** Striatal δ subunit expression at 2 months (*n* = 4) and at 6 months (*n* = 4). **(D)** α5 subunit expression in the striatum at 2 and 6 months in R6/1 mice (*n* = 4). **(E)** Striatal β3 subunit expression at 2 and 6 months in R6/1mice (*n* = 4). **(F)** Striatal β2 subunit expression at 2 and 6 months in R6/1mice (*n* = 4). ^∗^*p* < 0.05; ^∗∗^*p* < 0.01.

To evaluate GABAergic neurotransmission during HD progression, we quantified expression levels of several indicators essential for GABAergic synaptic function using Western blot on protein extracted from the striatum of mice at 2 and 6 months of age. The expression level of the GABA_A_R α1 subunit in R6/1 mice at 2 months was not modified statistically (WT: 100.0 ± 9.9; R6/1: 133.8 ± 22.6%) but was significantly increased (WT: 100.0 ± 3.5; R6/1: 159.9 ± 22.1%) at 6 months (**Figure 1A**). GABA_A_Rs present in synapses are associated with the γ2 subunit, while extrasynaptic receptors are mainly associated with GABA_A_Rs containing either α5 or δ subunits (Luscher et al., 2011). Expression of the γ2 subunit (**Figure 1B**) in R6/1 mice at 2 months (WT: 100.0 ± 1.4; R6/1: 108.2 ± 18.6%) and 6 months (WT: 100.0 ± 2.5; R6/1: 109.5 ± 8.1%) was not modified statistically, while the expression of the δ subunit showed a non-significant then significant decrease (WT: 100.0 ± 2.8; R6/1: 85.6 ± 8.2%, WT: 100.0 ± 2.1; R6/1: 67.4 ± 6.7%) at 2 and 6 months, respectively (**Figure 1C**). In direct contrast to δ, however, a non-significant change in α5 at 2 months (WT: 100.0 ± 4.9; R6/1: 145.8 ± 32.6%), became significant at 6 months (WT: 100.0 ± 3.7; R6/1: 196.8 ± 32.6%), in the R6/1 striatum (**Figure 1D**). GABA_A_Rs are pentameric complexes that contain β1-3 as mandatory subunits. Therefore, the expression of these subunits may reflect the expression level of GABA_A_Rs as a whole. The β3 subunit is the major subunit expressed in the striatum (Boyes and Bolam, 2007; Hortnagl et al., 2013). However, no change in expression level was detected for β3 at 2 or 6 months in R6/1 mice (**Figure 1E**). Furthermore, the β2 subunit, which is moderately expressed in the striatum, displayed an increased expression at 2 months that was then non-significant at 6 months (WT: 100.0 ± 2.9; R6/1: 152.1 ± 14.0% and WT: 100.0 ± 5.7; R6/1: 125.8 ± 20.0%, respectively; **Figure 1F**).

**FIGURE 2:**
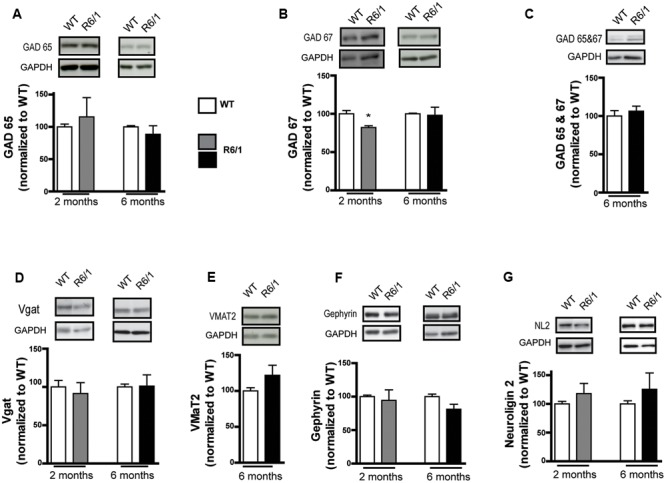
Western blot analysis of the striatum from 2 and 6 month-old mice with a pre-synaptic marker and adhesion protein antibodies. Striatal membrane preparations were subjected to SDS-polyacrylamide gel electrophoresis and immunoblotted. **(A)** Representative Western blots (from four) showing GAD 65 immunoreactivity (top panels). The bar graphs depict the means of four independent experiments and show GAD 65 expression after normalization to the corresponding expression of GAPDH; the mean ratios for 2 (gray bar, *n* = 4) and 6 month old R6/1 mice (black bar, *n* = 4) are displayed as a percentage of the corresponding wild type (WT, unfilled bars) taken as 100%. **(B)** Expression of GAD 67 in 2 and 6 month-old R6/1 mice (*n* = 4). **(C)** Western blot labeling with an antibody recognizing both GABA synthesizing enzymes, GAD 65 and 67, in the striatum of 6 month-old R6/1 mice (*n* = 4). **(D)** Vesicular transporter Vgat immunoreactivity in the striatum of 2 and 6 month-old mice (*n* = 4). **(E)** Expression of the vesicular transporter of monoamines, VMAT2, in 6 month-old R6/1 mice (*n* = 4). **(F)** Anchoring protein gephyrin immunoreactivity in the striatum of 2 (*n* = 4) and 6 month-old (*n* = 4) R6/1 mice. **(G)** Labeling with an antibody recognizing neuroligin 2 in the striatum of 2 and 6 month-old mice (*n* = 4). ^∗^*p* < 0.05.

Because GABAergic neurotransmission is also regulated by presynaptic mechanisms, the expression level of proteins involved in GABA synthesis or release was also measured. The level of GAD 65 was not altered in the striatum at 2 and 6 months in R6/1 (**Figure 2A**) while a significant decrease in GAD 67 levels was measured at 2 months (WT: 100.0 ± 4.4; R6/1: 82.1 ± 2.2%), but not at 6 months (**Figure 2B**). Consistent with these findings, no significant change was observed at 6 months with an antibody that recognizes both GAD 67 and GAD 65 (**Figure 2C**). Moreover, no significant changes in the expression of the vesicular transporter Vgat were observed (**Figure 2D**). Because GABA release by VMAT2-containing vesicles has been reported in the striatum (Tritsch et al., 2012), we measured the expression level of this vesicular monoamine transporter (**Figure 2E**); no significant changes were seen in the striatum of R6/1 mice. Finally, gephyrin and neuroligin 2 are predominantly associated with inhibitory synapses (Fritschy et al., 2008; Poulopoulos et al., 2009). The expression levels of these two proteins were not changed in the striatum of either 2 or 6 month-old R6/1 mice (**Figures 2F,G**).

**FIGURE 3:**
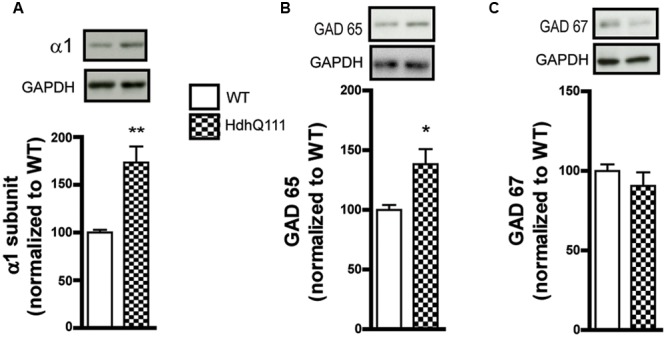
Western blot analysis of the striatum from 8 month-old HdhQ111 mice. Membrane preparations from 8 month-old WT and HdhQ11 mice were subjected to SDS-polyacrylamide gel electrophoresis and immunoblotted. **(A)** Representative Western blots of α1 subunit immunoreactivity with GAPDH as a loading control. The histograms depict the means of four independent experiments and show α1 subunit expression in HdhQ111 mice (hatched bar) after normalization to the expression of GAPDH and displayed as a percentage of the corresponding wild type (WT, unfilled bar) value. **(B,C)** Same figure layout as in **(A)** showing expression of GAD 65 **(B)** and GAD 67 **(C)**, respectively. Values are means plus SEM for 4 WT and 4 HdhQ111 performed in duplicate. ^∗^*p* < 0.05; ^∗∗^*p* < 0.01.

To corroborate our findings in the R6/1 model, we tested the HdhQ111 knock-in mouse line that is genetically distinct from R6/1 and has a different disease progression phenotype (Crook and Housman, 2011; Pouladi et al., 2013). Western blot analysis of membrane preparations extracted from the striatum of 8 month-old HdhQ111 mice revealed a statistically significant increase in GABA_A_R α1 subunit expression levels (WT: 100.0 ± 2.9; R6/1: 173.4 ± 16.8%, **Figure 3A**). In addition, the level of GAD 65 was significantly increased (WT: 100.0 ± 4.1; R6/1: 138.3 ± 12.6%) whereas the expression of GAD 67 was non-significantly reduced WT: 100.0 ± 4.1; R6/1: 98.5 ± 8.4%, **Figures 3B,C**). These results therefore are in accord with our data from the R6/1 mouse.

**FIGURE 4:**
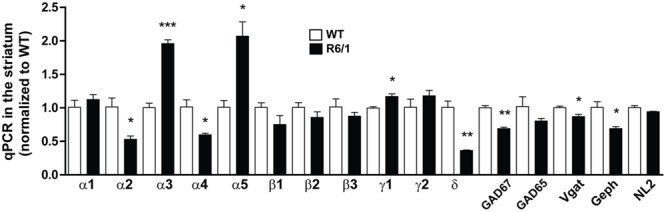
Quantitative PCR of mRNAs in striatal extracts from 6 month-old WT and R6/1 mice. The expression of mRNAs encoding for α1-5, δ, β1-3, and γ1-2 GABA_A_R subunits as well as GAD 65, GAD 67, Vgat, Gephyrin (Geph) and Neuroligin 2 (NL2) in 6 month-old WT and R6/1 mice were in each case as normalized to GAPDH and peptidylprolyl isomerase A expression. Mean values for R6/1 expressed as a percentage of corresponding WT are means plus SEM for 3 WT and 3 R6/1 performed in duplicate. ^∗^*p* < 0.05; ^∗∗^*p* < 0.01; ^∗∗∗^*p* < 0.001.

Protein expression analysis is an accurate way to assess gene expression downstream of transcriptional and translational regulation (Tian et al., 2004). However, Western blot is hampered by the limitation of finding suitable antibodies. Therefore, to validate and extend our findings, we performed quantitative PCR on mRNAs extracted from the striatum of 6 month-old WT and R6/1 mice (**Figure 4**). The significantly increased expression of striatal α1 subunit protein was not associated with a significant increase in mRNA expression, whereas the finding of a significant increase or decrease in the expression of α5 and δ mRNAs, respectively, was well correlated with our Western blot data (see **Figures 1C,D**). It should be noted that the mRNA of the α4 subunit that is associated with the δ subunit within extrasynaptic GABA_A_Rs was found to be also down-regulated in R6/1 mice. Interestingly, our q-PCR experiments showed that mRNA expression of α2 is decreased while the α3 subunit is increased in the striatum. Finally, the mRNA expression of the mandatory β subunits (either highly expressed β3 or the low-abundant β2 and β1 subunits) was not significantly modified. We also performed q-PCR on mRNAs encoding GAD 65, GAD 67, Vgat, neuroligin 2 and gephyrin extracted from the striatum (**Figure 4**). These experiments showed a decreased expression of GAD 67, Vgat and gephyrin mRNAs.

Taken together, our analyses of GABA_A_R subunit expression and pre- and post-synaptic protein markers of inhibitory synapses revealed subtle and complex changes in the striatum of R6/1 mice, with major alterations in α1, α2, and α3 subunit expression suggesting a change in the GABA_A_ receptor subtype. We therefore focussed attention on the expression of these three subunits in the striatum of WT and R6/1 mice.

### GABA_A_ α1 Subunit Expression in the Striatum Is Altered in MSNs and PV Neurons

**FIGURE 5:**
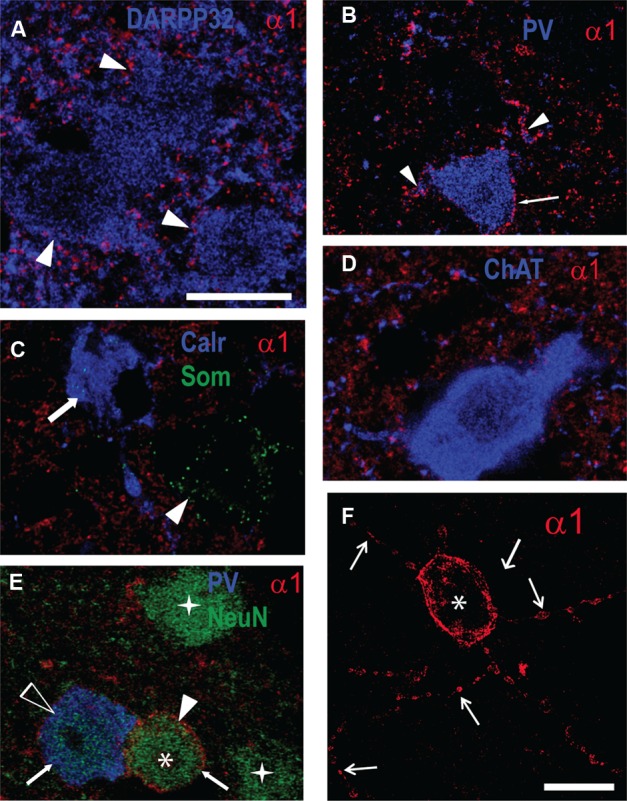
Fluorescent multiple-labelling of striatal neurons expressing the GABAAR a1 subunit. **(A–F)** Fluorescence labeling in sagittal sections of the striatum from WT mice for the GABA_A_R α1 subunit **(A–F)** along with DARPP32 **(A)**, parvalbumin (PV, **B,E**), calretinin and somatostatin (Calr and Som, **C**), choline acetyltransferase (ChAT, **D**), and NeuN **(E)**. PV interneuron cell membrane (**B**, arrow) and proximal dendrites (arrowheads) display α1 subunit labeling. Calretinin- (**C**, arrow) and somatostatin-containing cells (**C**, arrowhead) or cholinergic neurons **(D)** do not express α1 on their soma membranes. **(E)** Putative MSNs (stars) revealed by NeuN antibody staining, PV-positive neurons (unfilled arrowhead), and unidentified neurons (asterisk). Note the moderate α1 expression in the PV+ neuron compared to the high level in the unidentified neuron (arrows). **(F)** Single image projection created from a stack of 15 confocal z-sections (total depth 9.4 μm) of the unidentified striatal neuron expressing α1 displayed in (**E**; asterisk) showing dendrites with varicosities (arrows). Scale bars, 10 μm.

Our Western blot data described above showed an increase in GABA_A_R α1 subunit expression in the striatum of R6/1 mice. Since this subunit plays a major role in the kinetics of inhibitory post-synaptic currents (Luo et al., 2013), we performed multiple immuno-labeling on brain sections to assess its expression at the cellular level (**Figure 5**). Some staining was detected on MSN cell bodies revealed by DARPP 32 labeling, while major staining was found throughout the striatum, likely reflecting the subunit’s widespread distribution in the neuropil (**Figure 5A**). Parvalbumin (PV) interneurons were decorated with α1 puncta on their cell body membranes and proximal dendrites (**Figure 5B**). No co-localization with either calretinin or somatostatin dense-core vesicle labeling was detected (**Figure 5C**). Cholinergic neurons labeled by choline acetyltransferase (ChAT) antibodies did not display detectable α1 labeling (**Figure 5D**). In addition to the α1 labeling on PV interneurons, we also found a stronger labeling on NeuN positive-PV negative cells (**Figures 5E,F**). These neurons that expressed a high level of α1 are sparsely distributed (∼1 cell per section) and are characterized by a large dendritic tree displaying varicosities (**Figure 5F**). Labeling of 2 and 6 month-old R6/1 mice showed that this strong α1 staining on non-identified neurons was virtually absent in the mutant striatum.

**FIGURE 6:**
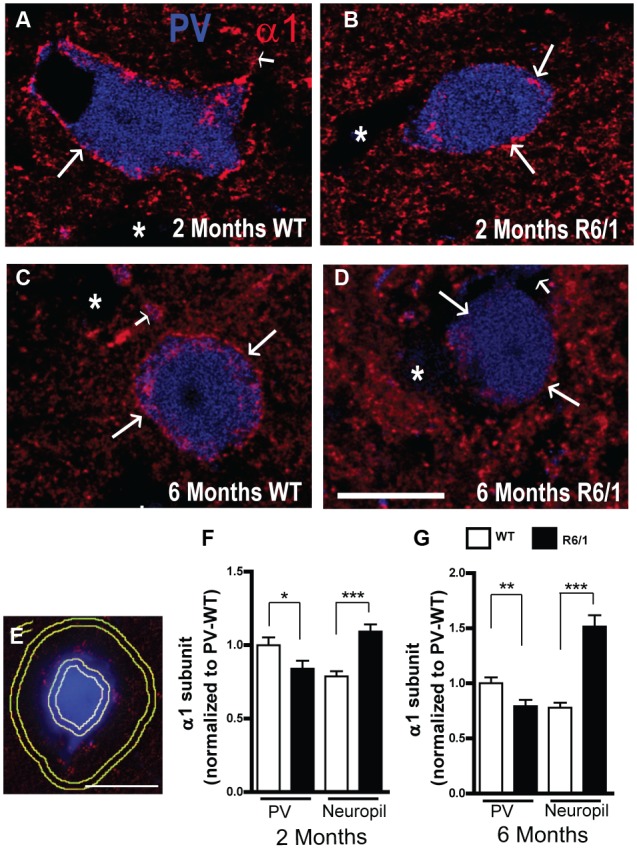
Quantitative analysis of GABAAR α1 subunit expression. **(A–D)** Representative confocal images showing fluorescent labeling for the GABA_A_R α1 subunit in the striatum of 2 **(A,B)** and 6 **(C,D)** month-old WT and R6/1 mice. Labeling is evident around PV+ cell bodies (arrows) and surrounding neuropil. Scale bar, 10 μm. **(E–G)** Analysis of the cell body and neuropilar region as depicted in **(E)** showing a significant decrease in α1 labeling on PV+ cells (*n* = 129–143 neurons for each age and genotype) and a significant increase on the neuropil **(F,G)**. ^∗^*p* < 0.05; ^∗∗^*p* < 0.01; ^∗∗∗^*p* < 0.001.

The expression of α1 on PV neurons and in the neuropil was further analyzed in striatal sections from 2 and 6 month-old mice (**Figure 6**). While fluorescent α1 labeling was clearly evident in confocal images of PV neurons from WT mice at 2 and 6 months (**Figures 6A,C**), it was less pronounced in R6/1 littermates at both 2 and 6 months (**Figures 6B,D**). To further assess this apparent overall decrease, the mean fluorescence intensity of α1 labeling was measured on the cell body periphery of PV-positive cells and on the surrounding neuropil (**Figure 6E**) and normalized to the corresponding expression values for PV positive cells from WT mice (**Figures 6F,G**). This quantitative analysis showed that a significant decrease in α1 subunit expression (83.8 ± 5.4% compared to WT PV cells) had indeed occurred on the somata of striatal PV positive interneurons in 2 month old R6/1 mice (**Figure 6F**). In contrast, the expression of neuropilar α1, which in WT was 78.7 ± 3.4% of the corresponding cell body expression, was increased significantly to 109.2 ± 4.8% in R6/1 mice (**Figure 6F**). Similarly at 6 months, α1 expression on PV neuron somata was decreased to 79.2 ± 5.8%, whereas the subunit’s presence in the neuropil, which in WT was 77.8 ± 4.4% of soma expression, had increased to 151.5 ± 10.2% in R6/1 mice (**Figure 6G**). Therefore, altogether these data support the conclusion that the increase in striatal α1 subunit presence in the R6/1 mutant revealed by Western blot analyses (**Figure 1A**) is due to an increased expression of α1 in MSNs, although intriguingly, this cell-specific increase is associated with an early decrease in the subunit’s expression in PV interneurons.

### GABA_A_ α2 Subunit Expression Is Altered in MSNs and PV and nNOS Interneurons

**FIGURE 7:**
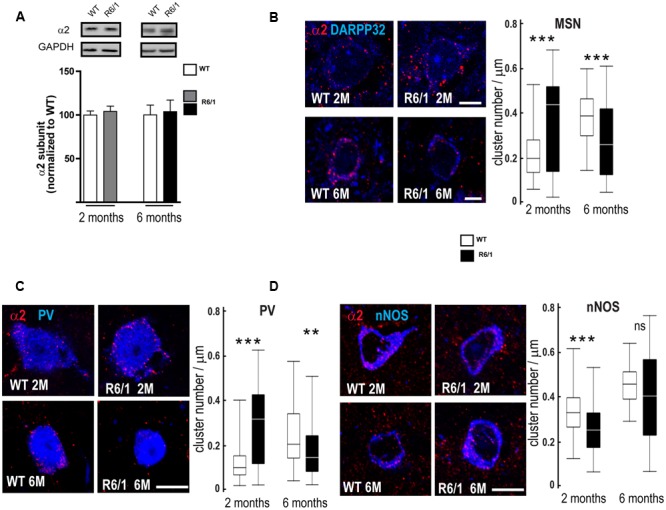
Quantitative analysis of GABAAR α2-subunit expression. **(A)** Western blot analysis of striatum extracts from 2 and 6 month-old WT and R6/1 mice. Top panel depicts representative Western blots (also probed for GAPDH as loading control) showing α2 subunit immunoreactivity. The bar graphs (Lower panel) depict the means of four independent experiments and show α2 subunit expression in R6/1 mice (gray bar, 2 months, black bar, 6 months) after normalization to the expression of GAPDH and calculated as a percentage of corresponding values for wild type (WT, unfilled bars) taken as 100%. **(B)** Representative confocal images showing fluorescent labeling for the GABA_A_R α2 subunit in the striatum of 2 and 6-month old mice. Labeling is evident around DARPP32+ MSN cell bodies and the surrounding neuropil. Scale bar, 5 μm. Box plots (central line: median; box: 25–75%; whiskers: min-max) at right quantify the increase or decrease in cluster density on MSN membrane at 2 and 6 months, respectively (*n* = 46–54 neurons for each age and genotype). **(C)** Images showing fluorescent labeling for the α2 subunit in the striatum of 2 and 6-month old mice. Labeling occurs around PV+ cell bodies. Scale bar, 10 μm. Box plots show the increase or decrease in cluster density on PV interneuron membrane at 2 and 6 months, respectively (*n* = 49–51 neurons for each age and genotype). **(D)** Images showing fluorescent labeling for the α2 subunit in the striatum of 2 and 6-month old mice. Labeling is evident around nNOS+ cell bodies. Scale bar, 10 μm. Box plots at right show the absence of any modification in cluster density on nNOS interneuron membrane at 2 and 6 months (*n* = 44–45 neurons for each age and genotype). ns: non-significant; ^∗∗^*p* < 0.01; ^∗∗∗^*p* < 0.001.

Our q-PCR analyses also indicated a decreased expression of the α2 subunit in the striatum of 2 and 6-month-old R6/1 mice (see **Figure 4**). However, subsequent Western blot comparison of striatal tissue protein extracted from WT and R6/1 mice did not indicate that significant subunit changes had occurred (**Figure 7A**). We therefore performed multiple immuno-labeling on brain sections to assess the expression of the α2 subunit at the cellular level. More precisely, striatal sections from 2 and 6 month-old WT and R6/1 mice were subjected to double-immunostaining in order to label the α2 subunit co-localized with dopamine- and cAMP-regulated neuronal phosphoprotein (DARPP32) that is only expressed in MSNs, or acetylcholinesterase, nitric oxide synthase and Calretinin that are specific to striatal GABAergic and non-GABAergic interneurons (Waldvogel et al., 1999; Ghiglieri et al., 2012; Calabresi et al., 2014). Staining of α2 was detected on MSN projection neurons, recognized by the presence of DARPP32, and GABAergic interneuron cell bodies located by co-staining with either PV or nNOS (**Figures 7B–D**). Subsequent analysis showed a significant increase of α2 subunit expression in striatal MSN and PV positive interneurons of R6/1 mice at 2 months and a significant expression decrease in these neuron subtypes at 6 months (**Figures 7B,C**). There was a significant decrease of α2 puncta in nNOS interneurons at 2 months, but no significant change was detected at 6 months (**Figure 7D**). These data thus suggest that, while the overall expression of α2 as revealed by Western blot analyses remains unchanged in the striatum of R6/1 mice, the subunit’s expression in specific neuronal subtypes is altered.

### GABA_A_ α3 Subunit Expression in the Striatum Is Altered in ChAT and PV Neurons

**FIGURE 8:**
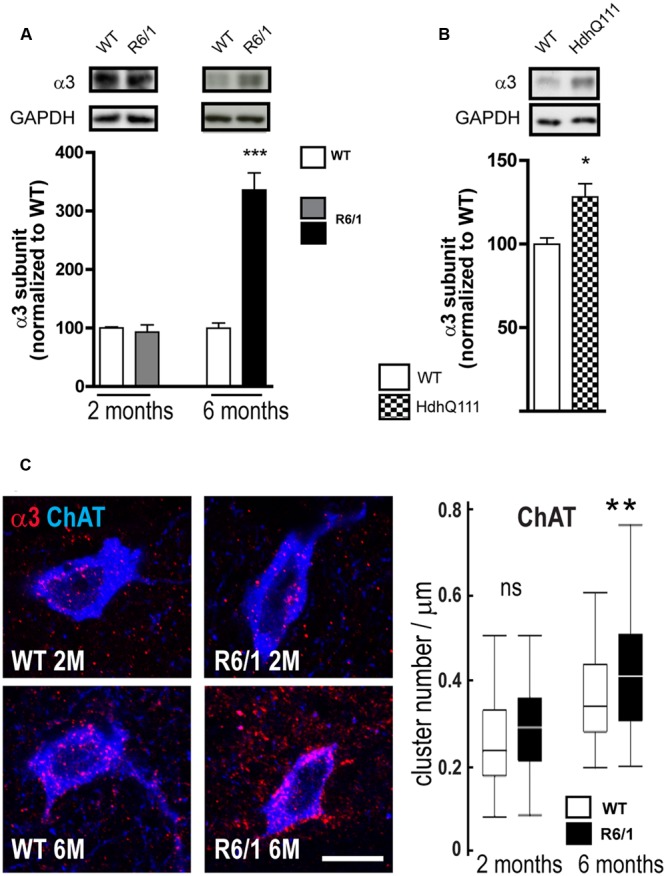
Quantitative analysis of GABAAR α3-subunit expression. **(A)** Western blot analysis of striatal extracts from 2 and 6 month-old WT and R6/1 mice. Representative blots (Top panel) show α3 subunit immunoreactivity. The bar graphs (Lower panel) depict the means of α3 subunit expression in R6/1 mice at 2 and 6 months (gray and black bars, respectively; *n* = 4 in each case) relative to GAPDH expression and normalization to corresponding wild type (WT) mean values set at 100%. **(B)** Expression of the α3 subunit in the striatum of 8 month-old HdhQ111 mice (*n* = 4). **(C)** Representative confocal images showing fluorescence labeling for the GABA_A_R α3 subunit in the striatum of 2 and 6-month old mice. Labeling is associated with cholinergic (ChAT-positive) cell bodies and surrounding neuropil. Scale bar, 20 μm. Box plots (central line: median; box: 25–75%; whiskers: min-max) at right show an absence of modification at 2 months and an increase in cluster density on neurons et 6 months (*n* = 52–59 neurons for each age and genotype). ns: non-significant; ^∗^*p* < 0.05; ^∗∗^*p* < 0.01; ^∗∗∗^*p* < 0.001.

Our q-PCR analyses also showed an increased expression of the α3 subunit in R6/1 mice. Correspondingly, Western blot analysis showed no significant change in R6/1 mice at 2 months and a significant increase (WT: 100.0 ± 8.4%; R6/1: 335.7 ± 29.5%) at 6 months (**Figure 8A**). Moreover, using the HdhQ111 knock-in mouse model, Western blot analysis of membrane preparations extracted from the striatum of 8-month-old mice also revealed a significant increase in the GABA_A_R α3 subunit expression level (WT: 100.0 ± 3.6; HdhQ111: 128.2 ± 7.9%, **Figure 8B**). We then performed multiple immuno-labeling of R6/1 striatal sections to assess the expression of α3 subunit at the cellular level. Staining was detected on interneuron cell bodies and proximal dendrites identified as cholinergic by Choline Acetyltransferase (ChAT) co-labeling (**Figure 8C**). Quantitative analysis (**Figure 8C**, right) showed that α3 subunit expression in such striatal interneurons is significantly enhanced at 6 months in R6/1 mice, suggesting that the subunit’s altered presence revealed by q-PCR and western blot analyses is due, at least in part, to an increased expression in cholinergic neurons.

### Altered Phenotypes of R6/1 Mice

**FIGURE 9:**
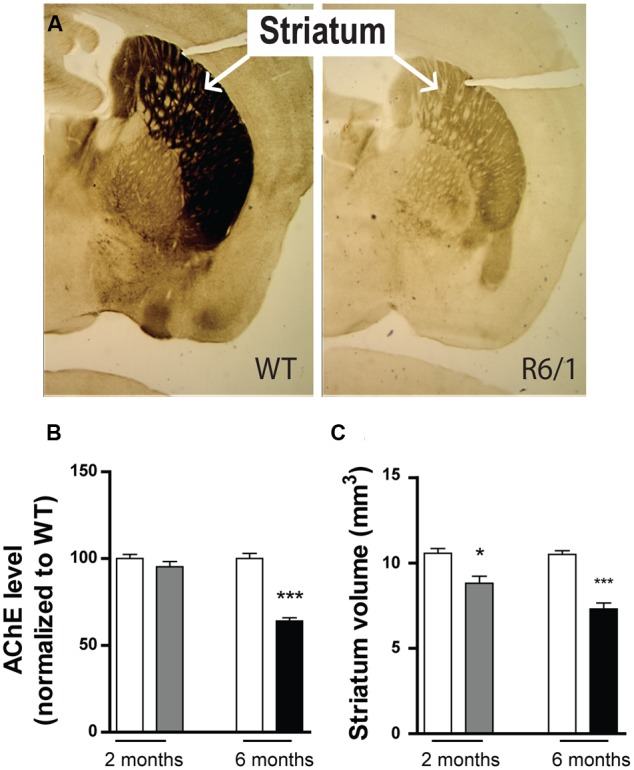
Phenotypic alteration in R6/1 mice. **(A)** Expression of striatal acetylcholinesterase in 6 month-old WT and R6/1 littermates. **(B)** Mean expression levels of acetylcholine esterase in the striatum at 2 (gray bar; *n* = 5) and 6 months (black bar; *n* = 3) relative to WT (unfilled bars). **(C)** Striatum volume calculations of 2 (*n* = 5) and 6 (*n* = 7) month-old WT and R6/1 littermates using acetylcholinesterase labeling ±SEM. ^∗^*p* < 0.05; ^∗∗^*p* < 0.01; ^∗∗∗^*p* < 0.001.

Because the expression of GABA_A_R subunits is already modified at 2 months in our R6/1 mice, we sought evidence to correlate this finding with actual brain pathology. Using acetylcholinesterase staining (**Figures 9A,B**), we found no change in acetylcholinesterase levels at 2 months (WT: 100 ± 2.4%; R6/1: 95.3 ± 3.0%; *n* = 5), but by 6 months, a dramatic reduction was evident (WT: 100.0 ± 3.3%; R6/1: 64.07 ± 1.86%; *n* = 3). Consistent with previous findings (Suzuki et al., 2001; Massouh et al., 2008), this is in turn indicative of a decline in acetylcholinesterase activity in the older animal and the resultant impairment of cholinergic neurotransmission. Additionally in R6/1 mice, striatal volume was significantly reduced at both 2 months (WT: 10.57 ± 0.28 mm^3^; R6/1: 8.81 ± 0.41 mm^3^; *n* = 5) and 6 months (WT: 10.50 ± 0.220 mm^3^; R6/1: 7.30 ± 0.355 mm^3^; *n* = 7) (**Figure 9C**). This concurs with magnetic resonance imaging data showing a decrease in R6/1 brain volume at 9 weeks (Rattray et al., 2013), and is also in agreement with recent observations on human HD showing that regional brain atrophy begins several years before the emergence of diagnosable symptoms (Ross et al., 2014).

## Discussion

**FIGURE 10:**
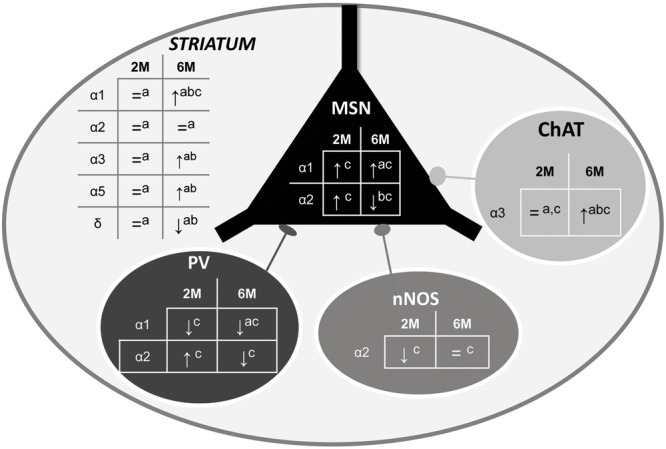
Summary of α1-, α2, and α3-GABAAR subunit expression in the overall striatum and in specific neuronal cell subtypes. The schematic representation of the striatum as a whole includes medium sized output neurons (MSN) and interneurons with parvalbumin (PV), nitric oxide synthase (nNOS), or Choline Acetyltransferase (ChAT). Expression alteration in the 5 locations in 2 and 6 month old R6/1 mice assessed by Western blot (a), q-PCR (b), or immunohistochemical (c) analyses are indicated.↑: increase, ↓: decrease, or = : no change.

The experiments reported here have led to several new findings showing that the HD pathology is associated with an alteration of the GABAergic system in the striatum. More precisely, using Western blot, q-PCR and immunohistochemistry, we show that the expression level of specific subunits at two different stages of the disease, is either decreased or increased depending on neuron subtype (**Figure 10**), indicating a change in GABA_A_R subtypes that would lead to a major alteration in inhibitory neurotransmission properties. Interestingly, these alterations were detected at an early age (2 months) when motor symptoms are still not expressed in our R6/1 mouse model of HD.

*Alteration of GABA_A_R subunit expression in the striatum*: Except for GAD67 expression in the striatum of 2 month-old R6/1 mice, we did not find significant changes in the expression of proteins involved in presynaptic structures or post-synaptic scaffolding. Strikingly, however, we found an increased expression of α1-, α3-, and α5- subunits and decreased expression of α4 and δ. It is noteworthy that, although the increased expression of α1-subunit protein was not confirmed by an associated increase in mRNA level, the increased expression of this subunit, as evidenced by Western blot analyses of striatal protein extracts, was further indicated by quantification of immunohistochemical labeling. On the other hand, the decreased α2-subunit mRNA level was not associated with a significant change in protein level. This is in agreement with many studies showing that following post-transcriptional and translational regulations, and degradation (Vogel and Marcotte, 2012), mRNA and protein levels are not always correlated (for examples, see (Ly et al., 2014). Nevertheless, we were able to correlate q-PCR and Western blot data for several proteins including the α3-, α5-, and δ-subunits. Our data are in line with recent studies that showed an upregulation of α3 and α5 mRNA expression as well as downregulation of α4 and δ (Achour et al., 2015; Langfelder et al., 2016). While the expression levels of some GABA_A_R subunits are altered in R6/1 mice, our data suggest that the overall quantity of GABA_A_Rs is not altered in the striatum of R6/1 mice. Firstly, Western blot analyses showed that the expression levels of gephyrin and NL2, both involved in inhibitory synapse formation (Luscher et al., 2011), are not altered. Second, Western blot or q-PCR analyses did not show dramatic alterations in the expression of mandatory GABA_A_R β-subunits. Third, Western blot analyses did not indicate a major change in the expression of α2-subunit, the principle α subunit in the striatum. Taken together, therefore, these findings indicate that the alteration of the GABA system in the striatum of R6/1 mice is specifically due to a change in GABA_A_R subtypes rather than the total number of receptors or synapses.

*Alteration of GABA_A_R subunit expression in MSNs:* It has been previously shown that the kinetics of GABAergic currents are altered in MSNs of animal models of HD (Cepeda et al., 2013), with a reduction both in rise and decay times leading to faster currents. Because it is well established that α1 is responsible for fast inhibitory currents (Vicini et al., 2001), the global increase of α1 subunits in the putative MSN neuropil supports a predominant contribution of the α1 subunit in IPSCs recorded from the MSNs of mutant mice. We also found an increase in α1 subunit expression in the striatum of HdhQ111 mice. This HD mouse model has been developed in a different genetic context to that of R6/1 and displays a mild phenotype and slow progression of the disease (Pouladi et al., 2013). These data are in line with a substantial increase of α1 immunofluorescent labeling reported in the striatum of 3 month-old R6/2 mice, when this model of HD displays overt symptoms (Cepeda et al., 2004). In addition to the increased expression of α1, immunohistochemical labeling of MSNs showed an increased expression of α2 at 2 months followed by a decreased expression at 6 months, although Western blot analyses did not reveal a significant change in the striatum as a whole. This apparent discrepancy suggests an alteration in GABA_A_R trafficking to the cell membrane and/or dendrites. The decreased expression of the α2 subunit in MSN cell bodies of 6 month-old R6/1 mice is consistent with a decreased number of GABAergic terminals in contact with MSN somata in the Z-Q175-KI mouse model of HD (Rothe et al., 2015). Interestingly, *in vitro* analyses have shown that cell surface expression of receptors as well as the expression of α1- and α2-subunits in MSNs are regulated by dopamine and GABA_A_R activity (Goffin et al., 2010; Arama et al., 2015). Thus, changes uncovered by the present study might be directly linked to alterations of both dopaminergic and GABAergic neurotransmission in HD (Cepeda et al., 2013; Francelle et al., 2014). It is also interesting that the α1 subunit is present in post-synaptic structures of dopaminergic striatal synapses (Uchigashima et al., 2016). Although these dopamine terminals are believed to release GABA (Tritsch et al., 2012, 2014; Nelson et al., 2014), their functional role remains elusive.

*Alteration of GABA_A_R subunit expression in interneurons:* Concomitantly with the increased α1 expression in MSNs, we found a decreased expression of this subunit in striatal PV interneuron cell bodies at 2 and 6 months while the expression of α2 is increased at 2 months and decreased at 6 months. GABAergic interneurons are involved in the coordination and regulation of network function in the brain, and many pathological conditions are linked to their alteration (Klaus et al., 2011; Gittis and Kreitzer, 2012; Calabresi et al., 2014; Ferando and Mody, 2014). In the striatum, it has been suggested that the functional role of inhibition from fast-spiking PV cells might be in shaping striatal output conveyed in both direct and indirect pathways (Planert et al., 2010). This feedforward inhibition from fast-spiking PV interneurons to MSNs is altered in HD (Cepeda et al., 2013). Because our findings suggest a modification of the GABAergic modulation of PV interneurons starting at an early stage in HD, a comprehensive study of the molecular, pharmacological and functional properties of GABAergic conductances in these cell types is now needed. The increased expression of α2-subunit and decreased expression of α1 in mutant mice predict that GABAergic currents in PV cells from 2 month-old R6/1 should have lower decay times compared to their WT counterparts (Vicini et al., 2001).

Cholinergic INs also play a major role in the physiology of the striatum (Pisani et al., 2007). It has been shown that in human and animal models, there is no, or a limited, loss of these INs, whereas the level of vesicular acetylcholine transporters or choline acetyl transferase is decreased (Suzuki et al., 2001; Smith et al., 2006; Massouh et al., 2008). In 6 month-old R6/1 mice, we show a decrease in acetylcholine esterase labeling, probably associated with a decrease in acetylcholine levels (Farrar et al., 2011). With immunohistochemical labeling, we found an increased expression of α3 GABA_A_R subunit in cholinergic INs. The α3 subunit is the main α subunit expressed in these striatal INs (Waldvogel et al., 1999) in which it is likely to represent the major GABA_A_R subtype. In R6/2 mice, compared to their WT counterparts, striatal cholinergic INs receive more GABAergic inhibitory post-synaptic currents, which may underlie the decreased level of acetylcholine in HD (Farrar et al., 2011; Holley et al., 2015). Consistent with this possibility, our data suggest that an increased number of post-synaptic GABA_A_Rs are involved in the increased inhibition of striatal cholinergic INs in HD.

*Alteration of extrasynaptic GABA_A_Rs expression:* Both δ- and α5-GABA_A_Rs are responsible for generating tonic inhibitory conductances in the brain, which is recognized as a key factor controlling local networks (Semyanov et al., 2004; Brickley and Mody, 2012). These two GABA_A_R subtypes are developmentally regulated in MSNs (Luo et al., 2013). A striking finding in our study was that α5 and δ subunit expression in the striatum is increased and decreased, respectively. It is of note that a decrease in striatal δ subunit-mRNA expression has been reported in different HD mouse models as well as in human patients (Kuhn et al., 2007; Seredenina and Luthi-Carter, 2012). As tonic inhibition is decreased in MSNs in mouse models of HD (Cepeda et al., 2013; Wojtowicz et al., 2013), our data suggest that a reduction in δ subunit expression plays a major role in this alteration. In addition to MSNs, the expression of both α5 and δ subunits has been identified in striatal INs (Schwarzer et al., 2001; Cepeda et al., 2013; Luo et al., 2013; Wojtowicz et al., 2013). Because tonic inhibition of interneurons may also be modified in many movement or psychiatric disorders (Gittis and Kreitzer, 2012; Ferando and Mody, 2014), it would be of interest to identify the neuron types whose specific α5 or δ subunit expression is modified in HD. It is also noteworthy that a clinical trial with an agonist of δ-containing GABA_A_Rs failed to improve symptoms of HD patients (Foster et al., 1983). On the basis of our findings, therefore, it would be relevant to test whether an α5-specific antagonist could improve disease progression or symptoms in HD (Mohler, 2011).

## Conclusion

Our data suggest that the overall level of GABAergic neurotransmission is not dramatically changed in the striatum of HD mice, even at a late stage in HD progression. Rather, we find that the pathology’s symptomatic progression is related to a change in the nature of GABA_A_Rs themselves. This is at odds with findings in the globus pallidus of R6/1 mice where the major alterations involve decreases in GABA_A_R subunits, synapses and inhibitory post-synaptic currents (Du et al., 2016). Interestingly, synaptic plasticity that regulates projection neurons in the cerebellum relies on interneuronal selectivity and on specific GABA_A_R subunit composition (He et al., 2015). Accordingly, our data provide the first molecular evidence supporting a profound synaptic plasticity in the striatum of a mouse model of HD which as a consequence may regulate the firing patterns of projection MSNs (Raymond et al., 2011; Ghiglieri et al., 2012). Importantly, this alteration is already significant at an early age, before the beginning of overt symptoms. In addition, increasing evidence indicates that abnormal development might play a role in HD (for review, see (Kerschbamer and Biagioli, 2015). Since subunit expression is developmentally regulated and changes in GABA_A_R subunit composition alter the outcome of GABAergic neurotransmission (Olsen and Sieghart, 2009; Luscher et al., 2011), we cannot exclude the possibility that differences in subunit expression between 2 month-old R6/1 mice and their WT counterparts are the consequences of an abnormal development.

## Author Contributions

ZD and MG designed research. YC supervised mouse breeding. ZD, MT, TL-L, LC, FM, and MG performed experiments. ZD, MT, GC, CH, and MG performed data analysis. ZD, CH, and MG designed all figures. MG wrote the manuscript with the help of other authors.

## Conflict of Interest Statement

The authors declare that the research was conducted in the absence of any commercial or financial relationships that could be construed as a potential conflict of interest.
